# Supplementary material for: Post-Mortem Extracorporeal Membrane Oxygenation Perfusion Rat Model: A Feasibility Study
Source: Animals (Basel). 2023 Nov 15;13(22):3532. doi: 10.3390/ani13223532 (PMC10668677; doi:10.3390/ani13223532)
Supplement: Supplementary file 1 [file animals-13-03532-s001.zip › Supplements.pdf]

Supplementary material

Post-Mortem Extracorporeal Membrane Oxygenation Perfusion Rat Model: A Feasibility Study

Matthias Manfred Deininger [1,2*]; Carl-Friedrich Benner [3]; Lasse Johannes Strudthoff [4]; Steffen Leonhardt [3]; Christian Simon Bruells [5]; Gernot Marx [1]; Christian Bleilevens [2] and Thomas Breuer [1]

Affiliations:

[1] Department of Intensive and Intermediate Care, Medical Faculty, RWTH Aachen University, Aachen, Germany

[2] Department of Anesthesiology, Medical Faculty, RWTH Aachen University, Aachen, Germany

[3] Chair for Medical Information Technology, Helmholtz-Institute for Biomedical Engineering, RWTH Aachen University, Aachen, Germany

[4] Department of Cardiovascular Engineering, Institute of Applied Medical Engineering, Medical Faculty, RWTH Aachen University, Aachen, Germany

[5] Department of Anesthesia, Intensive and Emergency Medicine, Marien Kliniken, Siegen, Germany

* Corresponding author: mdeininger@ukaachen.de

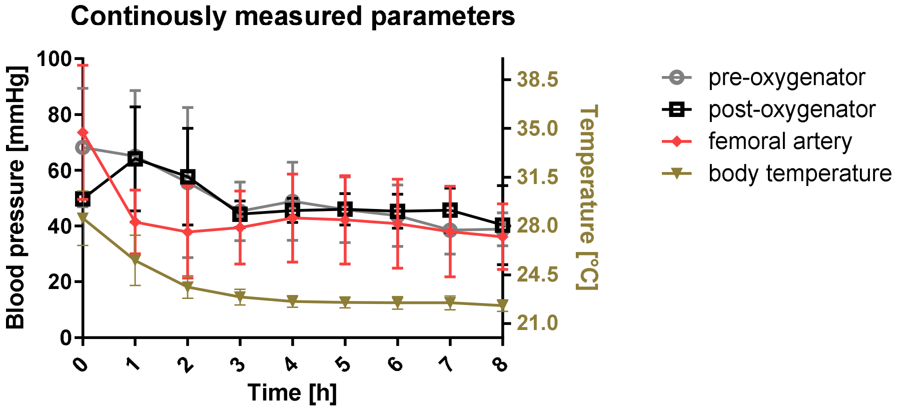

Figure S1. Mean blood pressure and temperature over the entire experimental duration

Data shown represent blood pressure/temperature averaged over one hour. In addition to femoral arterial blood pressure (femoral artery, red), extracorporeal blood pressures before (pre-oxygenator, grey) and after the oxygenator (post-oxygenator, black) were presented. The right ordinate shows the temperature (body temperature, brown) over time. Data are shown as mean ± SD.

Video S1: Angiographic organ perfusion

Sufficient organ perfusion was illustrated using angiography after arterial application of contrast medium bolus including arterial, portal-venous and venous phase.

Video S2: Digital subtraction angiographic perfusion

Organ perfusion was illustrated using digital subtraction angiography after arterial application of contrast medium including arterial, portal-venous and venous phase.
